# Direct observation of atomic-scale origins of local dissolution in Al-Cu-Mg alloys

**DOI:** 10.1038/srep39525

**Published:** 2016-12-21

**Authors:** B. Zhang, J. Wang, B. Wu, E. E. Oguzie, K. Luo, X. L. Ma

**Affiliations:** 1 Shenyang National Laboratory for Materials Science (SYNL), Institute of Metal Research, Chinese Academy of Sciences, Wenhua Road 72, 110016 Shenyang, China; 2 Electrochemistry and Materials Science Research Laboratory, Department of Chemistry, Federal University of Technology Owerri, PMB 1526 Owerri, Nigeria; 3 Guangxi Key Laboratory of Universities for Clean Metallurgy Comprehensive Utilization of Nonferrous Metal Resources, Guilin University of Technology, 541004 Guilin, China

## Abstract

Atomistic chemical inhomogeneities are anticipated to induce dissimilarities in surface potentials, which control corrosion initiation of alloys at the atomic scale. Precise understanding of corrosion is therefore hampered by lack of definite information describing how atomistic heterogeneities regulate the process. Here, using high-angle annular dark-field (HAADF) scanning transmission electron microscope (STEM) and electron energy loss spectroscopy (EELS) techniques, we systematically analyzed the Al_20_Cu_2_Mn_3_ second phase of 2024Al and successfully observed that atomic-scale segregation of Cu at defect sites induced preferential dissolution of the adjacent zones. We define an **“atomic-scale galvanic cell**”, composed of zones rich in Cu and its surrounding matrix. Our findings provide vital information linking atomic-scale microstructure and pitting mechanism, particularly for Al-Cu-Mg alloys. The resolution achieved also enables understanding of dealloying mechanisms and further streamlines our comprehension of the concept of general corrosion.

Dissimilar metals in electrical contact in the presence of an electrolyte undergo galvanic corrosion, in which one of the metals experiences an accelerated corrosion rate. This phenomenon is rather well known and has been extensively investigated and reported in the literature^1^,^2^,^3^,^4^,^5^,^6^,^7^,^8^,^9^,^10^,^11^,^12^,^13^,^14^. The galvanic corrosion is driven by the difference in open-circuit potentials of the galvanic couple. The important role of galvanic reactions in metal corrosion is well established and central to the understanding and control of metal corrosion^3^, the formation of protective coatings and the fabrication of nanostructured and porous metal structures^13^,^15^,^16^. The key concept is that electrons flow from anodic areas (with lower electrode potential) through the metal, to the cathodic areas, which accelerates corrosion in the anodic area. The same corrosion mechanism takes place on a microscale in the case of materials with heterogeneous microstructures, as typical of alloys having components with different noble character, such as age-hardened Al alloys^17^,^18^,^19^,^20^,^21^,^22^, duplex steels^23^,^24^,^25^, Mg alloys^26^,^27^,^28^,^29^ etc. The structural and chemical heterogeneity associated with second phase, precipitates or inclusions whose potential is differ from that of the surrounding matrix induces many miniature galvanic cells or “micro-galvanic cell” that could drive local electrochemical dissolution. Such local dissolution induced by micro-galvanic cells has been observed by means of optical microscopy, scanning electron microscope (SEM) and atomic force microscope (AFM). Usually, corrosion is recognized and understood at a relatively larger scale as above mentioned. However, infinitely close to the very initial stage, corrosion dissolution is believed to begin with departure of some atoms from the lattice position under the effect of the electrolyte. In a similar way, heterogeneities in chemistry and structure at the atomic level are expected to induce the initially preferential corrosion at atomic scale. Indeed, mesoscale and nanoscale theories of corrosion dissolution are now, more than ever before, relatively well understood, whereas there are significant knowledge gaps in our understanding of corrosion processes at atomic length scales. Without doubt the hypotheses that atom-scale defects could yield dissimilarities in surface potentials which induce preferential corrosion at the atomic scale are valid. Deciphering this link between microstructure and corrosion at the atomic scale will surely provide clear atomistic insights on the mechanism of the corrosion process. Unfortunately however, such an atomic-scale preferential dissolution has not yet to be observed directly.

Here, we use high-angle annular dark-field scanning TEM (HAADF-STEM) and electron energy loss spectroscopy (EELS) technique in TEM to visualize atomic-scale segregations at defect sites in the Al_20_Cu_2_Mn_3_ second phase of 2024Al, as well as preferential dissolution in the adjacent zones. We directly observe the role of “atomic-scale galvanic cells” in initiating local dissolution.

## Results

Our previous work identified the Al_20_Cu_2_Mn_3_ approximants as the initial dissolution sites in 2024Al. The dissolution process was inhomogeneous and occurred preferentially in the vicinity of the junction zone of multiple twins^30^. With this insight, we therefore set out to design an experimental procedure to facilitate close observation of the initial dissolution of the Al_20_Cu_2_Mn_3_ phase at the atomic scale and thereby attempt to establish an atomic-scale understanding of the link between microstructure and corrosion.

### Atomic-scale heterogeneity in chemistry triggers initial dissolution

We briefly immersed the 2024Al alloy in neutral 0.5 mol/L NaCl electrolyte, allowing enough time for very slight dissolution in the Al_20_Cu_2_Mn_3_ phase and then monitored the structural evolution using the HAADF-STEM technique in the TEM. The image contrast in such a mode is strongly associated with the local variation in chemical composition and/or thickness contribution^31^.

**Figure 1:**
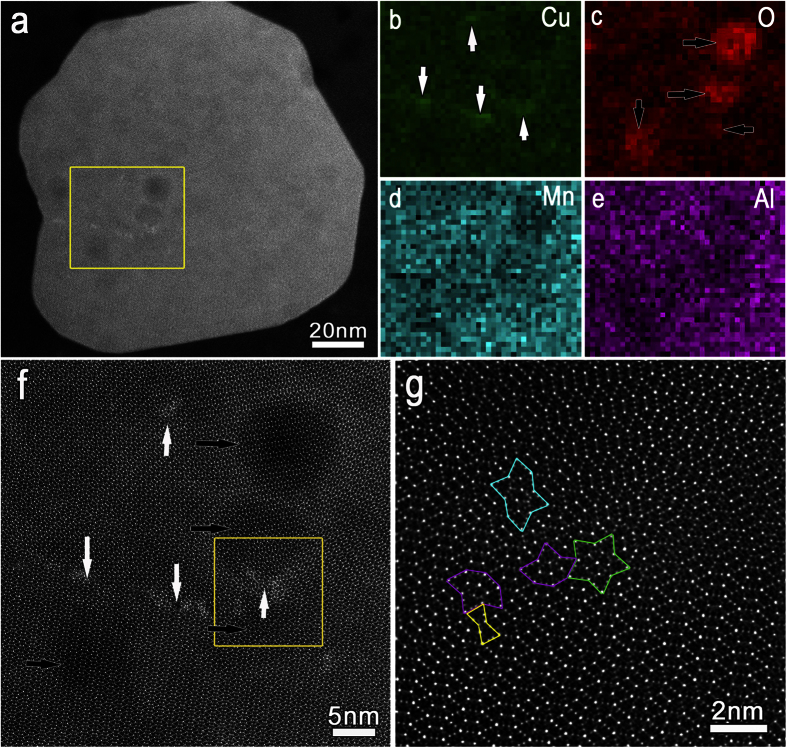
Heterogeneous chemistry induced local dissolution at the atomic scale: (**a**) HAADF-STEM image along the [010] axis showing an Al_20_Cu_2_Mn_3_ particle pre-immersed for about 8 min in 0.5 mol/L NaCl electrolyte. Slight dissolution occurs adjacent to the bright curled streak and a few dissolution sites with darker contrast encircled by the bright curled streak are obvious. (**b–e**) EELS mapping analysis focusing on the four spots enclosed within the box in (**a**). The darker spots are depleted in the elements Al, Mn, Cu and enriched in element O, while the brighter rings are enriched in Cu. (**f**) Magnified image corresponding to the boxed area in (**a**), highlighting the lattice and the hexagonal subunits beneath the corroded sites. (**g**) Magnified image of the boxed area in (**f**) showing a large number of defects within the Cu-rich streak.

Figure 1a illustrates the HAADF-STEM image along the [010] axis, showing a particle of Al_20_Cu_2_Mn_3_ immersed initially for about 8 min in neutral 0.5 mol/L NaCl electrolyte. A curled streak with brighter contrast is seen at a twin boundary. The bright streak has been previously determined to be enriched in Cu, with a large number of defects, as also shown here in Fig. 1f and g. Some small spots with slightly darker contrast are seen to be formed near the Cu-rich streak and each dark spot is bordered by a ring with brighter contrast. Observations from EELS mapping analysis of the four spots within the rectangle in Fig. 1a are shown in Fig. 1b–e. It is obvious that the darker spots are depleted in the elements Al, Mn, Cu and enriched in O, while the brighter rings are enriched in Cu, as previously established^30^. All of these indicate slight dissolution within the dark spots, to yield copper-rich products at the ring sites bordering the spots. Similar character of Cu element redistribution after corrosion was detected at relatively larger scale in our previous work^30^. We were quite fortunate that the ultrathin corrosion products from the slight dissolution of Al_20_Cu_2_Mn_3_ allowed us to directly observe the lattice and the hexagonal subunits immediately beneath the corroded areas, as shown in the high resolution HAADF-STEM image (Fig. 1f). Interestingly, the result confirms, as widely believed, that it is at the atomic scale that dissolution ensues. Preferential dissolution evidently originates from atomic-scale heterogeneities in chemistry and microstructure within the Al_20_Cu_2_Mn_3_ phase. The bright curled streak, formed by the segregation of Cu at atomic-scale defects, and its adjacent zone constitute a galvanic couple, in which the nobler Cu-rich zone triggered preferential dissolution of the adjacent zones. We are bold to term this system an **“Atomic-scale galvanic Cell**”, akin to the concept of the micro-galvanic cell.

Interestingly, geometric phase analysis (GPA) of strain distribution in the vicinity of the Cu-rich defects revealed no contribution from strain-induced dissolution (Supplementary Text and Supplementary figures S1 and S2), thus confirming that initial corrosion dissolution originated strictly from atomistic dissimilarities in surface potentials, induced by the atomic-scale galvanic cells.

We observed the position of the corrosion initiation at atomic scale and are bold to present an idea of “atomic-scale galvanic cells” first time. Macroscopically, a galvanic cell is generally understood to involve an anodic reaction taking place on one part of a metal with a cathodic reaction taking place on another part of a metal. The thermodynamic driving force is the difference of electrode potential between various parts of a metal, where the region with the lower potential is the anode suffering corrosion. Certainly, the corrosion rate is determined by the dynamic factors. As an example of 2024Al alloy, usually at the initial stage, θ phase (Al_2_Cu) and Al matrix act as the cathodic place on which the ORR (oxygen reduction reaction) occurs in neutral electrolyte, and S (Al_2_CuMg) phase has been generally known as the anodic place on which Al and Mg undergo the selective dissolution. However, as reported in our previous work, the position of initial dissolution doesn’t locate at the interface of S (Al_2_CuMg) phase/Al matrix but governed by an additional nano-galvanic cell of S (Al_2_CuMg) phase– T (Al_20_Cu_2_Mn_3_) phase. Accurately, the nano-galvanic cell is comprised by S (Al_2_CuMg) phase and the Cu-rich remnant from dissolution of Al_20_Cu_2_Mn_3_. The detailed anodic and cathodic process has been described in our previous work^30^. The large amounts of nano-galvanic cells determine the initial corrosion locations of S phase and are also crucial to the activity of S phase. In the same manner, for the T (Al_20_Cu_2_Mn_3_) phase, apart from the cathodic Al matrix and S phase, the additional galvanic cell of T phase/Cu-rich twin boundary is crucial which determines the activity of T phase and the initial dissolution position. Although the scale of the Cu-rich twin boundary is atomic, it still can act as cathodic place on which the ORR occurs. Certainly, the cathodic ORR can occur on the other cathodic places simultaneously. Dynamically, the fastest anodic reaction takes place at adjacent to the Cu-rich twin boundary. **Since the Cu-rich boundary is at atomic scale, we are bold to define the couple of T phase/Cu-rich as “atomic scale galvanic cells”.** In other words, even in an anodic-role particle with nanometer scale, the potential distribution is still heterogeneous. The surrounding “large-scale cathodic circumstance” has almost identical effect on the nano-particle, however, the atomic scale defects in the interior have the crucial effect determining the dynamic process. The experimental phenomenon^30^ that the T phases without rotated twins or with multiple twins but free of Cu-rich boundaries behave inactive and are free of corrosion at the initial corrosion stage, further proves the importance of the “atomic scale galvanic cell”. Under the effect of the atomic scale galvanic cell, the region adjacent to the Cu-rich line undergoes the initial corrosion where the selective dissolution of Al and Mn is involved. In other words, the T phase indeed undergoes the selective dissolution, whereas, the location where the selective dissolution begins is determined by the atomic scale galvanic cell.

It is noteworthy that the dissolution did not initiate at the site immediately adjacent to the Cu-rich streak, but rather at locations several decades of atomic layers away from the streak. We believe the interface between the Cu-rich zone and the adjacent zone to be in the form of a transition layer (or atomic diffuse layer), wherein the Cu content decreases very gradually away from the streak, yielding a weak difference in contrast that is difficult to distinguish. Accordingly, the location with lowest Cu content, and hence with most negative potential, where the dissolution will occur preferentially, will be far removed (by several atomic layers) from the Cu-rich zone. Interestingly, from Fig. 1a and f we observe that the bright Cu-rich rings link up in a manner depicting the figure 8- (i.e. 8-shaped). Contrariwise, the O-rich zones do not touch each other and are only situated within the dark spots, as shown in Fig. 1c, implying that oxides are principally formed at the center. It is therefore very likely that the Cu-rich ring is comprised of elemental copper, not copper oxide.

Accordingly, we propose the following mechanism for the dissolution of Al_20_Cu_2_Mn_3_: The atomic-scale galvanic cells induce preferential dissolution of Al and Mn, with some hydrolyzed ions subsequently redeposited as oxides. In order to specify the particular oxides, we considered the E-pH diagrams of Mn/ H_2_O and Al/ H_2_O. According to the E-pH diagrams, the formation of manganese hydroxide by hydrolysis is not thermodynamically feasible in neutral NaCl solution (pH = 7), whereas formation of aluminum hydroxide is feasible. Again, the available E-pH diagram for Mn-H_2_O shows that manganese hydroxide can only be formed in strongly basic electrolytes. Aluminum, on the other hand, is amphoteric and aluminum hydroxide is stable at pH 4–9, therefore, the hydrolysis of aluminum ions can occur readily under the test conditions. From the foregoing, it is clear that dissolution of Mn would not yield the hydroxide or oxide, the O-rich centers should thus be ascribed to the hydrolysis of dissolved Al ions. Moreover, the hydrolysis is expected to induce weakly acidic conditions locally, which could still sustain hydrolysis of Al ions.

**Figure 2:**
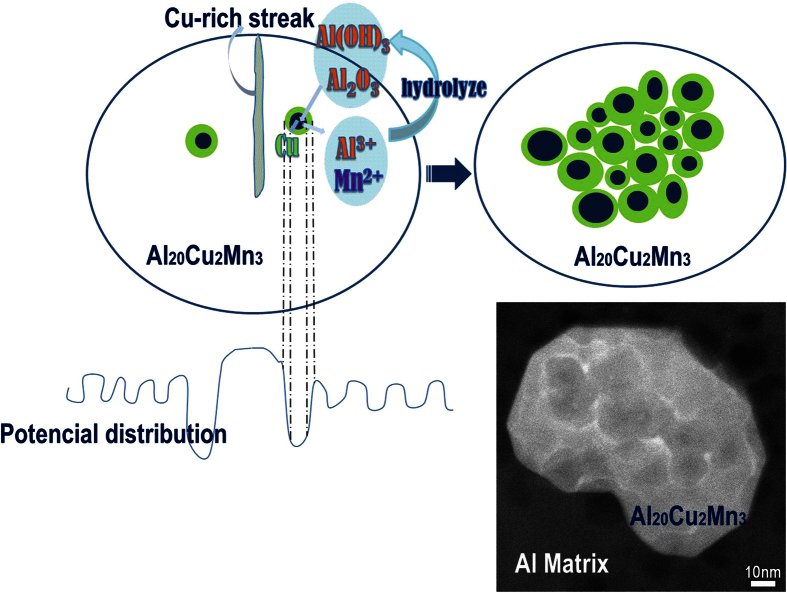
Propagation of dissolution: (**a**) Schematic illustration depicting the atomic scale dissolution of Al_20_Cu_2_Mn_3_ as well as the formation of the Cu-rich rings, arising from atomic scale fluctuations in potential distribution. The potential drop reaches a maximum at some site near the Cu-rich dislocations, where dissolution initiates. As a result, Cu-rich rings are preferentially formed at the periphery of the dissolution sites. Hydrolysis of Al^3+^ inhibits further dissolution and at the same time there is potential ennoblement at the newly formed Cu-rich ring. Both effects trigger fresh dissolution at an adjacent location, which is subsequently propagated over the entire surface as illustrated in (**b**). The HAADF image in (**c**) shows the morphology of the dissolved Al_20_Cu_2_Mn_3_ particle, which is consistent with the sketch (**b**).

The Cu-rich ring is obviously formed as a result of dealloying. This means that the ability to image and observe dissolution at the atomic scale also allows new insights that will increase our understanding of the dealloying process. Dealloying has been creatively harnessed in the fabrication of porous metals and recently extended to production of nanoporous^32^ or nanostructured metal oxides^33^,^34^,^35^. For aluminum alloys, a number of studies rightly observed that the second phase materials in aluminum alloys dissolve selectively, with the active components dissolving preferentially to leave a Cu-rich remnant. Such studies however were unable to provide sufficiently detailed information on the plausible mechanisms of the process; hence no rational explanation has been postulated to describe the actual mechanism of the Cu redistribution. Interestingly, our findings suggest two possible paths for the redistribution. Firstly, it is likely that the selective dissolution of Al and Mn caused the Cu adatoms to become unstable, with tendency to diffuse outwards via surface or volume diffusion processes, to form the bright Cu-rich rings at the periphery of the dissolution sites (dark spots), as shown in Fig. 1a and f. The other possible path considers the atomic-scale galvanic couples arising from the potential distribution at the surface, as illustrated in Fig. 2a, where the copper ions from the dissolution sites are reduced to copper at the ring sites. This reasoning is supported by the E-pH diagram of the Cu-Cl-H_2_O system, where the corrosion product of Cu in 0.5 mol/L NaCl is soluble copper chloride at pH < 9 and Cu_2_O at pH > 9. Accordingly, the dissolution of Cu within the active site is also permitted thermodynamically in the neutral environment, whereas the formation of Cu_2_O as reaction product is not feasible. Certainly both paths describing Cu redistribution may occur simultaneously and are in fact accommodated in the *surface or volume diffusion* as well as the *dissolution-redeposition* mechanisms that have been proposed in the realm of porous copper/copper oxide preparation^32^.

### Atomic-scale heterogeneity and propagation of dissolution

It is clear that hydrolysis of the released Al ions will inhibit further action at the initial dissolution sites, while aggregation of Cu leads to potential ennoblement around the ring sites. Both effects trigger new dissolution at an adjacent site and this in turn to the next adjacent site. Proliferation of this process as illustrated schematically in Fig. 2a, propagates the Cu-ringed dissolution sites all through the entire surface. Accordingly, the morphology of the dissolved Al_20_Cu_2_Mn_3_ particle should then comprise of several dissolution sites bordered by the rings, as illustrated in Fig. 2b. Interestingly, our obtained HAADF-STEM image of the dissolved Al_20_Cu_2_Mn_3_ particle very clearly supports such a mechanism (Fig. 2c), revealing several dark spots (dissolution sites) within brighter Cu-rich rings, which is consistent with the sketch in Fig. 2b. We can imagine that such an intricately elaborate morphology, cannot be observed at a larger-than-atomic scale, but would rather be obscured by a Cu-rich residue, following the selective dissolution of the Al and Mn components. This actually validates the long-postulated concept that corrosion dissolution is initiated at the atomic-scale.

**Figure 3:**
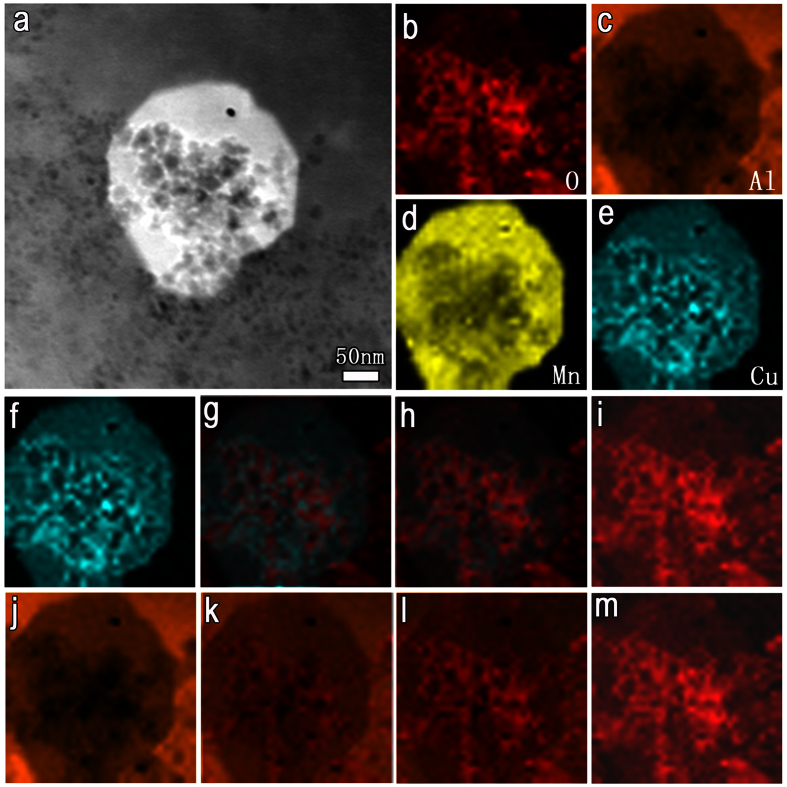
Elemental redistribution within the Al_20_Cu_2_Mn_3_ particle, where localized dissolution occurs. (**a**) HAADF-STEM image showing a more severely dissolved Al_20_Cu_2_Mn_3_ particle. Lots of small pits with darker contrast and the mesh frame with brighter contrast are left behind. (**b–e**) EELS mapping analysis on the particle; the darker pits are depleted in Al and Mn while the brighter mesh frame is enriched in Cu. (**f–i**) Composite graphs obtained by superimposing the O map (red) on the Cu map (blue) with variant opacity of 0% (**f**), 30% (**g**), 70% (**h**) and 100% (**i**). The frames enriched in Cu and O do not overlap, signifying that the Cu-rich frame is not the copper oxide. (**j–m**) The composite image obtained by superimposing the O map (red) on the Al map (orange) with variant opacity of 0% (**j**), 20% (**k**), 60% (**l**) and 100% (**m**). Both frames overlap, confirming the formation of aluminum oxide. This implies possible redistribution of aluminum oxide into aggregates and clusters.

Figure 3 shows the HAADF-STEM image and the EELS mapping results of another particle which has undergone relatively more severe dissolution. It is immediately obvious that the character of the corrosion morphology is identical to that at the initial corrosion stage, particularly vis-à-vis the dark dissolution sites encircled by the brighter Cu-rich rings. However, from the EELS mapping results (Fig. 3b–e), the texture of the mesh frame for elemental O no longer correspond with the darker corrosion pits, but is rather analogous to that of the Cu frame. This effect could either be ascribed to the redistribution of aluminum oxide with progress of the corrosion reaction or to the formation of copper oxide at this stage of corrosion. In order to clarify this, we superimposed the O map on the Cu map, while varying the opacity to obtain composite graphs as shown in Fig. 3f–i. The frames enriched in Cu and O did not overlap, but were just staggered with respect to each other, indicating that the Cu-rich frame is not the oxide. Another composite image was obtained by superimposing the O map on the Al map, wherein the frames overlapped each other, implying formation of aluminum oxide. This means that redistribution of aluminum oxide into aggregates and clusters occurred as the severity of corrosion increased. Although we did actually detect copper oxide (Cu_2_O) when the Al_20_Cu_2_Mn_3_ and the adjacent S phase dissolved almost completely and the corrosion propagated to the Al matrix (Supplementary figure S3), the corrosion product at the initial stages was copper, not copper oxide.

## Discussion

The in-depth insights derived from investigating the initiation and propagation of corrosion at the atomic level is of great significance for very precise understanding and control of the associated electrochemical dissolution processes. The present work also highlights the possibility of employing analytical methods with very high spatial and mass resolution for deciphering atomic-scale dissolution processes. An important implication of our findings has to do with the widely accepted concept of general/uniform corrosion, which may actually not exist in an absolute sense, when atomic-scale galvanic cells are taken into consideration.

## Methods

### Sample preparation

2024 aluminum alloy with nominal composition of Al–4.35Cu–1.55 Mg–0.53Mn–0.11Fe–0.13Si-0.03Ti (wt.%) was chosen for this investigation. Details of the preparation and treatment procedures are as outlined in our previous work^30^. Briefly, solution treatments were performed at 495 °C, after which the alloy was cold-drawn into a tube at room temperature with a shrinkage of 8–15%, and then annealed at 380 °C for one hour. A linear precision saw was used to cut the sample into 600 μm-thick sections, perpendicular to the deformation direction. These were subsequently ground to about 100 μm thickness using 1000 grit silicon carbide papers. Disks with diameter of 3 mm were prepared by die-cutting, then ground using variant grit silicon carbide papers, polished with diamond paste to 20 μm, and finally thinned by ion-milling. The TEM specimens were immersed into the neutral 0.5 mol/L NaCl solution at room temperature for various periods (the duration ranged from 8 to 25 min), then were retrieved and very carefully cleaned in distilled water, dried, plasma-cleaned and then transferred into the TEM for investigation.

### TEM and STEM characterization

A Titan G2 60–300 spherical aberration corrected transmission electron microscope (Cs-corrected TEM) was used at 300 kV for high-angle annular dark-field scanning TEM (HAADF-STEM) imaging and composition analysis. This microscope is fitted with a high-brightness field-emission gun (X-FEG), double Cs corrector from CEOS, and a monochromator (with a point resolution of 0.7 Å), and equipped with a high-angle annular dark-field (HAADF) detector and electron energy loss spectrometer (EELS) systems. The beam convergence angle is 25 mrad, and thus yields a probe size of less than 0.10 nm.

### Strain analysis by Geometric Phase Analysis (GPA)

Custom plugins for GPA by Gatan Digital Micrograph were used to analyze strain distribution in locations near the Cu-rich defects, where corrosion dissolution initiates. Piecewise analysis was performed, in which each twin segment was cut accurately along the edges of twin boundaries and subsequently extracted and made ready for GPA map analysis (Supplementary figure S2a–b). The reference lattice was selected within the interior of the segment. All single maps of the twin segments were finally spliced together to yield a composite map.

## Additional Information

**How to cite this article**: Zhang, B. *et al*. Direct observation of atomic-scale origins of local dissolution in Al-Cu-Mg alloys. *Sci. Rep.* **6**, 39525; doi: 10.1038/srep39525 (2016).

**Publisher's note:** Springer Nature remains neutral with regard to jurisdictional claims in published maps and institutional affiliations.

## Supplementary Material

Supplementary Information
