# Supplementary material for: Direct observation of atomic-scale origins of local dissolution in Al-Cu-Mg alloys
Source: Sci Rep. 2016 Dec 21;6:39525. doi: 10.1038/srep39525 (PMC5175146; doi:10.1038/srep39525)
Supplement: Supplementary Information [file srep39525-s1.pdf]

Direct observation of atomic-scale origins of local dissolution in Al-Cu-Mg alloys

B. Zhang[1*†], J. Wang[1*], B. Wu[1], E. E. Oguzie[2], K. Luo[3], X. L. Ma[1†]

Supplementary Text

Discuss on the strain state within the twin segments

Twins or multiple twins occur frequently in the orthorhombic $Al_{20}Cu_2Mn_3$ approximant of decagonal quasicrystal. The rotation of twins in $Al_{20}Cu_2Mn_3$ is close to, but not exactly 36 °. Therefore, the $Al_{20}Cu_2Mn_3$ hardly forms perfect tenfold twins with ten twin domains, but rather forms one or more irregular twin boundaries at the junction zone, with large amounts of defects, where Cu segregates (Supplementary figure S1 a-c). Such imperfect tenfold twins should normally have some associated strain. Few particles free of twins (d, e) or with twins but no Cu segregation can also exist.

As we know, local strains can induce local dissimilarities in potential, which could as well result in preferential electrochemical dissolution. In order to distinguish initial corrosion dissolution associated with atomic-scale heterogeneities in chemistry from strain-induced dissolution near the Cu-rich defects, we used the ***Custom plugins for geometric phase analysis (GPA), by Gatan Digital Micrograph*** to analyze the strain distribution in $Al_{20}Cu_2Mn_3$.

The GPA is an effective approach to determine crystal lattice variations within a large area of a high-resolution HAADF-STEM image, and thus deduce the strain distribution state. The deformations in each twin segment were determined piecewise and relative to an internal reference lattice. The piecewise analysis[1] enabled us to uniquely determine the strain state within the segments, but limits our ability to address sharp changes at the boundaries between the segments. This, gratifyingly, is adequately sufficient for our immediate objective, particularly since dissolution initiates at the interior of the twin segments and not at the twin boundaries. The results of the GPA analysis of an $Al_{20}Cu_2Mn_3$ particle is shown in Supplementary figure S2. Supplementary figure S2d shows the composite maps obtained by splicing together all single maps of the twin segments. The homogeneous contrast of the composite map means that no strain exists near the defects and confirms that our observed initial corrosion dissolution originated from atomic-scale heterogeneities in chemistry and not from strain-induced dissolution near the Cu-rich defects.

We wish to point out that the two streaks appearing in segment 1 and one streak in segment 2, with evidently dissimilar contrasts as seen in the GPA map (Supplementary figure S2 d) are in no way associated with the strain gradients. From the zoom-in, high resolution HAADF-STEM image (Supplementary figure S2 c), the three strips marked with green lines, corresponding to the location of the dissimilar streaks in the GPA map, clearly coincide with the rotated array of the hexagon subunits. Therefore, the dissimilar contrasts originated from the rotated array and

definitely not from any strain gradients. Moreover, the areas with the rotated hexagons are too small to be cut as a separate segment for GPA analysis.

Supplementary figures

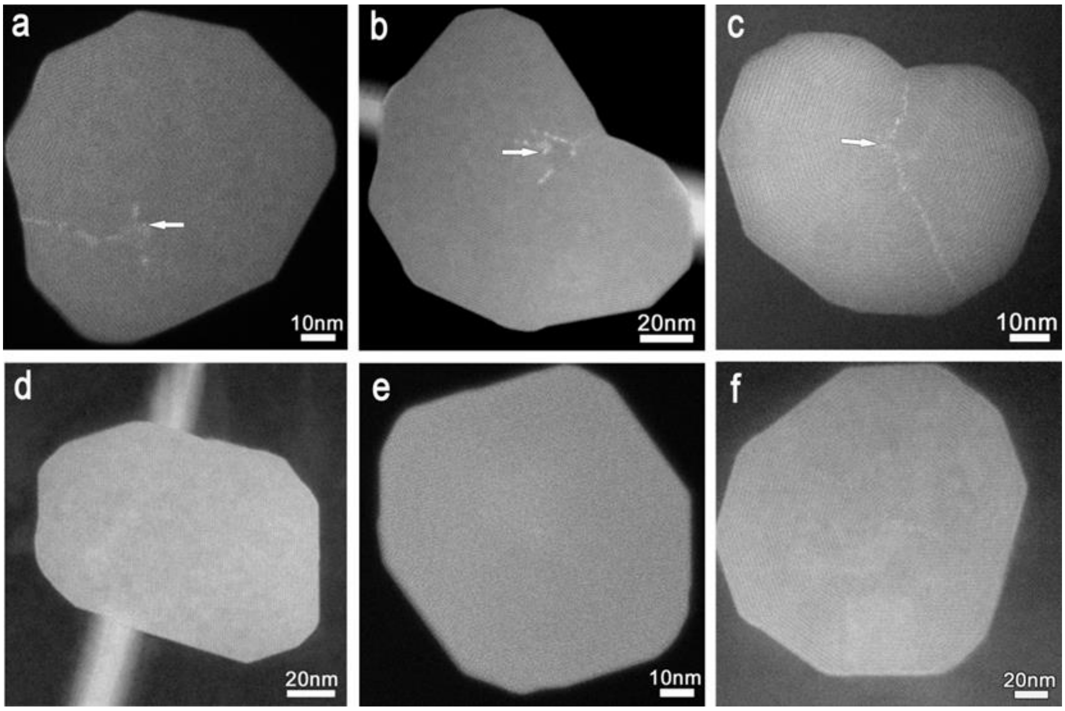

Supplementary figure S1. HAADF-STEM images along the [010] axis showing two types of $Al_{20}Cu_2Mn_3$ particles classified with reference to Cu segregation: (a-c) with Cu segregation and (d-f) without Cu segregation.

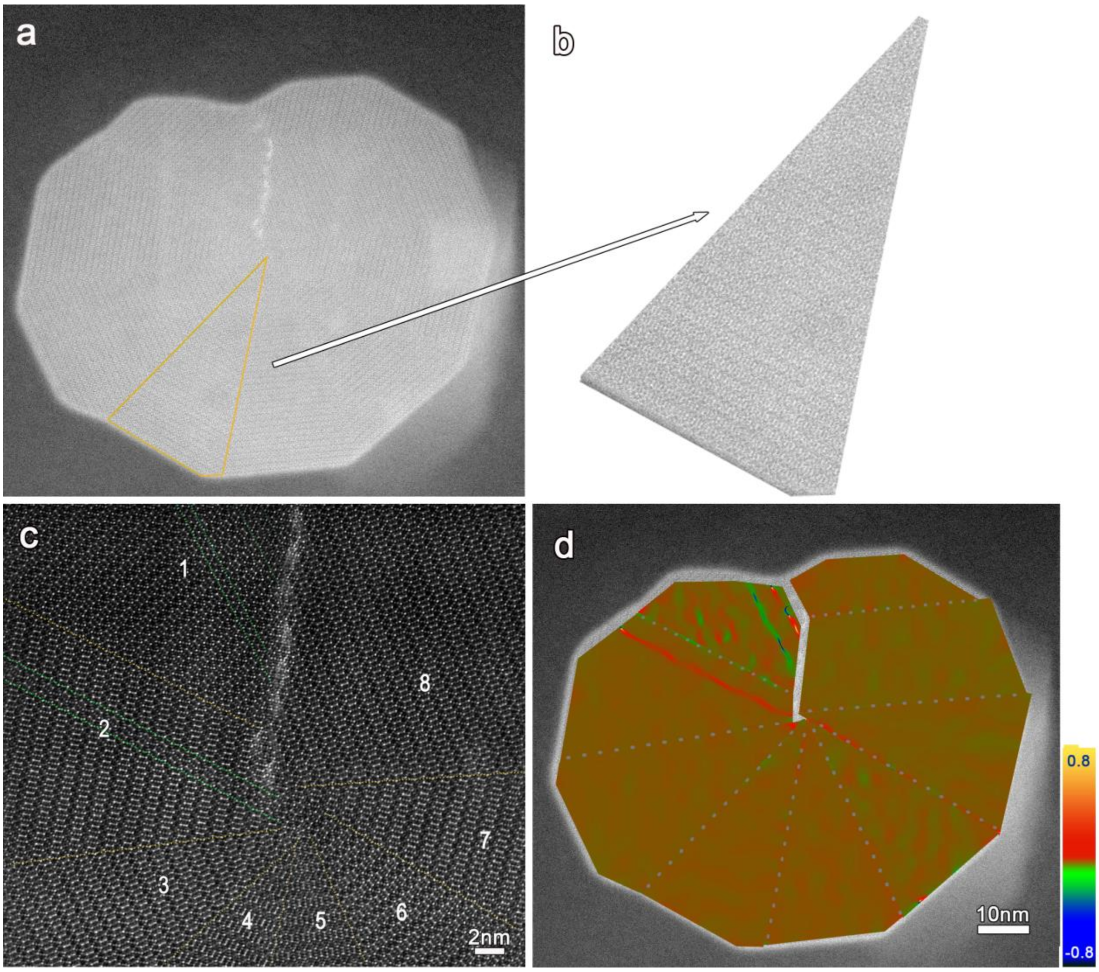

Supplementary figure S2. GPA analysis on the STEM data, showing that there are no strains existing in the twin segments of $Al_{20}Cu_2Mn_3$ phase**. (a)** HAADF-STEM image along the [010] axis showing an $Al_{20}Cu_2Mn_3$ particle with rotated multiple twins. **(b) A representative segment, cut along the twin boundaries, ready for GPA analysis. (c)** The zoom-in high resolution HAADF-STEM image of (a). The yellow lines are boundaries along which several segments were cut for GPA analysis. (d) Composite GPA maps obtained by splicing together all the single maps of the twin segments; the homogeneous contrast is indicative of the absence of strain near the defects.

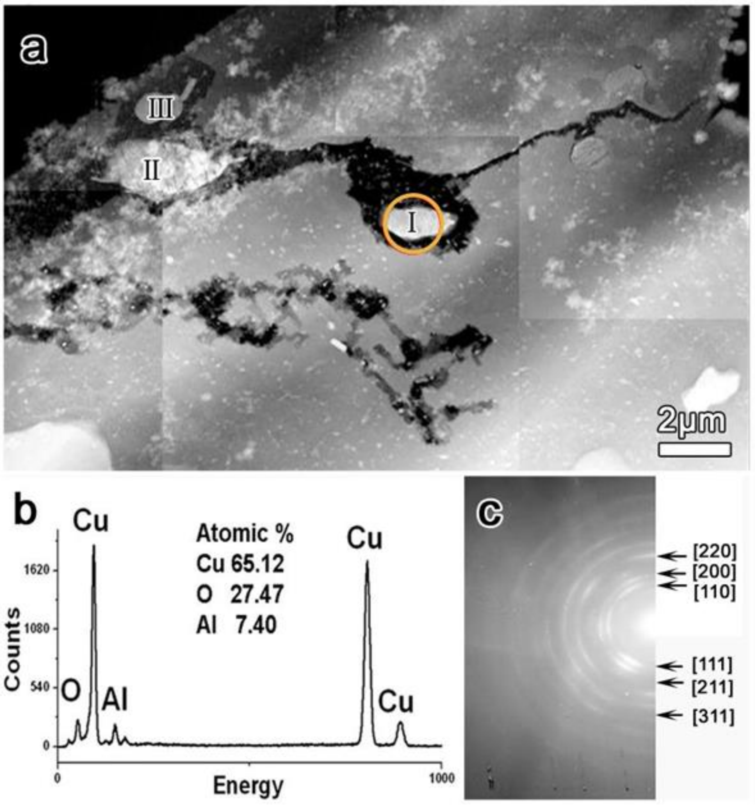

Supplementary figure S3. Cu_2O product is formed when the $Al_{20}Cu_2Mn_3$ and the adjacent S phase dissolved almost completely and the corrosion propagated to the Al matrix. (a) The TEM image shows the corrosion morphology of 2024Al. (b) EDS analysis of the severely dissolved particle labeled by I within the circle. The EDS profiles include the well-defined Cu peaks, evident O peak and weak Al peak, which indicates element Al has been selectively dissolved almost completely and a Cu-rich corrosion product is formed. (c) The EDP obtained from the particle I, which identifies the Cu_2O as the main product.

References:

[1] Johnson, C. L., Snoeck, E., Ezcurdia, M., Rodriguez-Gonzalez, B., Pastoriza-Santos, I., Liz-Marzan, L. M. & Hytch, M. J. Effects of elastic anisotropy on strain distributions in decahedral gold nanoparticles. Nat. Mater. 7, 120-124 (2008).
